# Topical Insulin in Neurotrophic Keratopathy: A Review of Current Understanding of the Mechanism of Action and Therapeutic Approach

**DOI:** 10.3390/pharmaceutics16010015

**Published:** 2023-12-20

**Authors:** Marcin Jaworski, Anna Lorenc, Rafał Leszczyński, Ewa Mrukwa-Kominek

**Affiliations:** 1 Department of Ophthalmology, Kornel Gibiński University Clinical Center, Medical University of Silesia, 40-752 Katowice, Poland; 2 OPTOMED Ophthalmological Center for Children and Adults, 41-500 Chorzów, Poland; 3 Department of Ophthalmology, Faculty of Medical Sciences in Katowice, Medical University of Silesia, 40-752 Katowice, Poland

**Keywords:** topical insulin, corneal ulcer, neurotrophic keratopathy, eye disease, corneal insulin receptors

## Abstract

Neurotrophic keratopathy is a corneal disease characterized by impaired corneal innervation. It can lead to corneal epithelial defects, ulcerations, and perforations. Topical insulin has been shown to be effective in treating this disorder. Insulin is a growth factor that can promote corneal epithelial cell proliferation and migration. In addition, it can also inhibit corneal epithelial cell apoptosis. Topical insulin has previously been found to enhance corneal wound healing. This article reviews the current understanding of the mechanism of action of topical insulin in the treatment of neurotrophic keratopathy.

## 1. Introduction

Neurotrophic keratopathy is a rare corneal disorder, with an estimated prevalence of less than 5/10,000. The incidence of neurotrophic keratopathy is notable among patients presenting with corneal ulcers, with rates between 13% and 27% [1]. While the primary focus of insulin administration has historically centered on its role in the treatment and management of diabetes, its potential therapeutic effects in other fields have also been a subject of investigation.

The year 2021 marked the 100th anniversary of the discovery of insulin by Banting and Best. The discovery of this hormone forever changed the face of medicine and the treatment of diabetes. It influenced the development of molecular biology, physiology, and the understanding of the mechanism of prohormone processing [2]. Insulin is an anabolic peptide hormone that mediates metabolic processes and regulates cell growth and proliferation. It consists of two chains: an A chain and a B chain, linked by disulfide bonds. Insulin is produced in β cells in the pancreas. Its production is controlled by feedback depending on glucose and insulin levels [3].

There are 14 types of glucose transporters (GLUTs), which constitute the primary method of glucose uptake by cells in mammals [4].

The vast majority of cells require insulin for glucose uptake. For example, in skeletal muscle cells and adipocytes, it binds to INSR and promotes glucose uptake from the circulation via glucose transporter-4 (GLUT4). The corneal epithelium shows that glucose uptake occurs via the GLUT1 transporter and does not require the presence of insulin. The number of transporters can increase when metabolic demand increases, such as as a result of corneal injury [5].

In a pioneering study conducted in 1945, researchers explored this hormone’s capacity to aid in the healing of corneal ulcers, a condition that can severely and irreversibly compromise visual acuity and even lead to blindness. This early research highlighted improvement in corneal ulcer healing after systemic administration of insulin, setting a precedent for further studies to explore its potential applications in ophthalmology, and posing both diagnostic and therapeutic dilemmas for ophthalmologists [1]. Following the initial study that highlighted the potential of insulin to improve corneal ulcer healing, additional research in this area stalled. It was not until the 1990s that interest in the therapeutic applications of insulin in ophthalmology was rekindled. Over the past two decades, there has been a significant uptick in the topical use of insulin in corneal ulcer treatment, partly driven by the higher incidence of diabetes and ophthalmic surgeries (such as posterior segment vitrectomy) that can lead to the onset of neurotrophic keratopathy and subsequent severe corneal ulcers.

In humans, there is a single gene responsible for insulin production—INS—located on chromosome 11. Its transcription is controlled by factors IDX1, MafA, NeuroD1, and many other coregulators. In contrast, rodents have two genes, ins1 and ins2. This reveals that the mechanisms controlling secretion, as well as presumably metabolic pathways, differ in humans and other organisms. Animal studies and results can vary from species to species [6].

Neurotrophic keratopathy is characterized by impaired corneal innervation. A pathognomonic symptom of neurotrophic keratopathy is corneal hypoesthesia, which denotes diminished corneal sensation. This impairment can arise following viral corneal infections (e.g., HSV, VZV) in the context of systemic diseases (such as diabetes mellitus and multiple sclerosis) or can be due to iatrogenic causes such as corneal surgeries and posterior vitrectomies (Table 1 and Table 2) [7,8]. Regardless of the cause, however, the clinical severity of neurotrophic keratopathy remains poorly elucidated, especially in terms of restoring corneal sensation [8].

Alterations in corneal nerve function compromise both the sensation and the structure of the cornea, leading to a series of detrimental changes. These changes can include deficiencies in neuropeptides, neurotransmitters, and growth factors, which manifest as abnormal tear production, diminished blinking reflex, loss of protective reflex, intracellular corneal edema, and desquamation of epithelial cells. Furthermore, there is a notable reduction in the corneal goblet cell count. Cumulatively, these changes result in epithelial instability and impede wound healing, potentially progressing to corneal ulcerations and, in advanced stages, tissue perforation. Thus, compromised innervation directly affects the health and stability of the tear film, corneal epithelium, and stroma [14].

Diabetes, particularly, has a significant impact on the cornea. It induces tear film instability due to reduced density of conjunctival goblet cells and decreased production by the lacrimal and meibomian glands. Additionally, there is damage to corneal neurons, which diminishes corneal sensation and the frequency of blinking, leading to further damage. Several mechanisms of damage are distinguished, including the accumulation of advanced glycation end products (AGEs) that accumulate in the proteins of peripheral neuron myelin. The sorbitol–aldose reductase pathway leads to osmotic stress of neural cells during hyperglycemia. Neuronal damage also occurs as a result of oxidative stress. The activation of the protein kinase C (PKC) pathway during hyperglycemia contributes to the deterioration of the neuronal function [15,16].

Neurotrophic keratopathy progression occurs in three stages. Some researchers proposed substages.

### 1.1. Staging Proposed by Mackie et al. (1995) [8,17]

1. Stage I of neurotrophic keratopathy is characterized by punctate epithelial staining and punctate keratopathy, indicating localized areas of epithelial cell damage. There is also evidence of hyperplasia and irregularity in the corneal epithelium, along with superficial neovascularization. This stage is further defined by a decreased tear film break-up time and Rose Bengal staining of the inferior palpebral conjunctiva, highlighting areas of cellular damage or dryness. Gaule spots are noted, representing scattered areas of dried epithelium. Additionally, there is an increase in mucous tear viscosity.
2. Stage II is characterized by a persistent epithelial defect, typically located on the superior half of the cornea, surrounded by a rim of loose epithelium. The edges of the defect may smoothen and can become loose or roll, leading to further deterioration. There may also be stromal edema present, indicating swelling within the corneal stroma. An anterior chamber inflammatory reaction is rare but may occur.
3. Stage III is the most severe form, presenting with a corneal ulcer, where there is a deeper loss of the corneal tissue. There is also stromal melting, a serious condition where the corneal stroma breaks down rapidly, and potentially, perforation of the cornea [8,17].

### 1.2. Staging Proposed by Dua et al. (2018) [14]

1. Stage I (mild) is characterized by superficial epithelial punctate lesions, alterations in the tear film, and reduced or absent sensitivity in isolated or multiple regions of the cornea.
2. Stage II (moderate) presents with persistent epithelial defects and may include partial to complete loss of corneal sensation.
3. Stage III (severe) manifests with stromal involvement, potentially progressing to ulcerations or full-thickness perforations, alongside diminished to entirely absent corneal sensation [14].

### 1.3. Staging Proposed by Mastropasqua et al. (2019) [11]

1. Stage I
  - Stage IA of neurotrophic keratopathy is identified by a punctate epithelial fluorescein stain, which may occur with or without corneal hypoesthesia. Diagnostic imaging shows that sub-basal nerve fiber density is equal to or greater than 5 μm/mm^2^, and total nerve fibers are equal to or greater than 1 μm/mm^2^. Anterior-segment optical coherence tomography reveals an irregular epithelial surface, increased epithelial and anterior stromal reflectivity, while stromal thickness and Bowman’s layer are preserved.
  - Stage IB is distinguished by a sub-basal nerve fiber density of less than or equal to 5 μm/mm^2^ and total nerve fibers of less than or equal to 1 μm/mm^2^.
2. Stage II
  - In Stage IIA, there is an epithelial defect with smooth or rolled edges and corneal hypoesthesia or anesthesia. The sub-basal nerve fiber density is equal to or greater than 3 μm/mm^2^. Optical coherence tomography findings include an interrupted epithelial layer and increased anterior stromal reflectivity, without corneal stroma thinning or ulceration.
  - Stage IIB is characterized by a sub-basal nerve fiber density of less than or equal to 3 μm/mm^2^.
3. Stage III
  - Stage IIIA presents corneal ulceration with stromal thinning, where the thickness depth is equal to or less than 50%.
  - Stage IIIB is marked by stromal thinning and/or ulceration with a thickness depth of 50% or greater [11].

Therapeutic approaches are contingent upon the stage of progression [8,14]. With standard therapy, 60% of cases take longer than 14 days to heal, and approximately 50% heal completely [13].

Insulin was discovered in 1921, following years of research to find a treatment for diabetes [3]. Insulin is a polypeptide essential for cell proliferation and metabolism regulation, and it is considered to be the main anabolic hormone in the body. This hormone is detectable in the tear film [18], and its impact on the corneal epithelium is likely mediated through insulin receptors present on ocular surface structures. Based on existing research, topical application of insulin may be effective in treating certain anterior segment eye conditions. This article reviews the current understanding of the mechanism of action of topical insulin in the treatment of neurotrophic keratopathy.

## 2. Cellular and Molecular Mechanisms of Neurotrophic Keratopahy

The cornea, with its remarkable density of nerve endings, stands as the most exquisitely sensitive tissue within the human body, containing 11,000 nerve endings per square millimeter. These nerves, an extension of the ophthalmic branch of the fifth cranial nerve, are fundamental to ocular protective mechanisms, including tear secretion, the blink reflex, and the healing of corneal wounds. Furthermore, the cornea’s integrity is sustained by its cells. The corneal epithelium’s nerve endings are sensitive to temperature, pain, and pressure, and respond to diverse stimuli by releasing neuromediators that preserve the cornea’s structural integrity.

Afferent corneal innervation orchestrates lacrimation and palpebral reflexes, contributing to ocular hydration and protection, respectively. This innervation is pivotal for nociception and the maintenance of corneal health, facilitating tissue repair. The corneal epithelium, densely covered with nerve endings from the ophthalmic division of the trigeminal nerve (V1), encompasses an extensive neural network within its stroma, basal epithelium, and sub-basal nerve plexus. The nasociliary branch of V1 courses through the orbit to the corneal surface, and multiple ciliary nerves intersect, perforate the sclera, and radiate through the corneoscleral limbus into the stroma. Corneal innervation patterns, confirmed by in vivo confocal microscopy and histological staining, exhibit an even nerve distribution along the corneal circumference. The stromal neural architecture, composed of thick myelinated A-delta and thin unmyelinated C fibers, discards myelin sheaths within the stroma to preserve corneal transparency. These fibers diverge throughout the stroma, with some innervating keratocytes and others advancing to form the subepithelial plexus. Eventually, these fibers perforate the Bowman’s membrane, establishing the sub-basal nerve plexus, predominantly made up of C fibers. This plexus is instrumental in epithelial sustenance, promoting cell growth and differentiation by releasing neuromediators such as calcitonin gene-related peptide (CGRP), acetylcholine (Ach), and substance P (SP) [9,10,11,12,13].

The primary symptom of neurotrophic keratopathy is a compromised corneal sensation, leading to subsequent disruptions in tear film stability, blink reflex, and ultimately, corneal damage. The lacrimal nerve, a V1 branch, is responsible for the sympathetic innervation that governs tear secretion, while the parasympathetic innervation of the facial nerve (cranial nerve VII) increases tear production. It is essential to consider the additional lacrimal and meibomian glands, which are innervated differently [10,11,12].

The tear film is a stratified entity, beginning with an aqueous mucin phase marked by a gradient of concentration and topped with a complex lipid layer. The inner mucin layer, abundant in mucin (MUC)-5AC from conjunctival goblet cells, ensures an even distribution of the aqueous layer by creating a hydrophilic surface. The corneal and conjunctival epithelial cells produce mucins such as MUC-1, MUC4, and MUC-16, contributing to the glycocalyx—a gel-like coating on the cellular microvilli that supports the aqueous mucin phase of the tear film [9].

The tear film’s aqueous layer, its largest constituent, is a reservoir of vital substances like electrolytes, proteins essential to the innate immune defense, secretory IgA, lysozyme, lactoferrin, and growth factors that promote epithelial cell health, predominantly secreted by the principal lacrimal gland and supplemented by accessory glands. These glands are connected to efferent fibers stemming from V1 through the pterygopalatine ganglion.

The cornea is innervated by three primary types of neurons, each with distinct functions:

- Polymodal nociceptors are the most abundant type, responding to a variety of noxious stimuli including mechanical, thermal, and chemical insults. They play a key role in the sensation of pain and triggering reflexive protective responses such as blinking and tearing.
- Mechanonociceptors, which comprise a smaller percentage of corneal neurons, are specialized to respond exclusively to mechanical forces that are potentially harmful enough to damage the corneal surface. These neurons are responsible for the initial pain sensation in response to mechanical stimulation.
- Cold thermoreceptors, the least numerous, are activated by a decrease in corneal temperature, typically due to tear evaporation. These receptors contribute to the sensation of ocular surface dryness and discomfort and can initiate reflex tearing to restore corneal hydration.

All receptors contribute to sensations of pain, discomfort from polymodal stimuli, dryness, and cooling from cold receptors.

Corneal nerves express a variety of neuromediators that, apart from their direct effects, also influence tear secretion, which in turn carries growth factors nourishing the cornea. Following damage, afferent corneal nerves release inflammatory mediators, initiating a neurogenic inflammatory response. These neuromediators, present in both somatosensory and autonomic fibers, foster a conducive environment for ocular surface repair, stimulating epithelial cell proliferation, migration, adhesion, and differentiation. Keratocytes, residing within the stroma, either transform to adopt a fibroblastic repair phenotype or undergo apoptosis in response to injury. Epithelial–stromal interactions, regulated by TGF-β2 released by epithelial cells, control this process, which relies on an intact epithelial basement membrane. A compromised membrane can lead to fibrosis rather than regenerative repair. Keratocytes also express neuromediators and their receptors, which promote wound healing. Sensory nerve fibers, specifically polymodal nociceptors, and their neuromediators, such as substance P and its receptors (NK-1R), neurokinin A (NKA) and receptors (NK-2R), CGRP and receptors (CRLR), galanin and receptors (GALR1-3), and pituitary adenylate cyclase-activating peptide (PACAP) with its receptors (VPAC1R, VPAC2R, and PAC1R), play significant roles in epithelial regeneration and wound healing, as well as corneal sensation post-injury [9].

Acetylcholine, as a result of the activation of the parasympathetic system, may have a beneficial effect on corneal healing. Acetylcholine accelerates mitosis in corneal epithelial cells, enhancing proliferation. In mice, the application of atropine, a muscarinic acetylcholine receptors antagonist, increased neutrophil infiltration and proinflammatory cytokines in injured corneas, hindering reepithelialization after abrasion. These findings suggest that muscarinic acetylcholine receptors activation could potentially mitigate neutrophil recruitment and thereby improve healing and epithelization [9].

## 3. Dosage and Treatment Efficacy

The available literature presents a wide range of insulin concentrations utilized in the treatment of corneal ulcers occurring in conjunction with neurotrophic keratopathy from 0.5 to 100 IU of short-acting or regular insulin per 1 mL of polyethylene glycol solution or saline. Initially, in the 1990s and early 2000s, high concentrations of insulin (25–100 IU/mL) were used. Even at high doses up to 100 IU/mL, insulin application was found to be safe [19,20]. The safety of higher concentrations indicates that they do not cause harm, but it does not imply greater efficacy. The current preference for lower concentrations likely stems from proven efficacy in healing at lower doses, combined with a precautionary approach to minimize drug exposure and any potential, albeit low, toxicity. Thus, recent publications increasingly favor lower concentrations, typically 0.5–1 IU/mL [19,21,22,23,24,25]. This significant reduction in concentration still achieves clinical efficacy, but with presumably reduced drug toxicity [19]. Dasrilsyah et al. [19] found that topical insulin at a dosage of 0.5 units administered four times daily is superior in healing corneal epithelial defects in diabetic post-vitrectomy patients, compared with placebo or higher insulin concentrations (1 or 2 IU/mL).

In addition, insulin is more effective in epithelial restoration than autologous serum drops [26], which has primarily been shown by case series reports [19,21,22,23,24,25]. The dosage used in these reports varied, typically one drop administered 2–4 times daily [19,20]. In animal models, insulin application may enhance corneal sensation, potentially offering effects similar to surgical corneal neurotization methods. However, larger trials using standardized corneal sensation measurement methods are needed [27,28,29].

**Figure 1:**
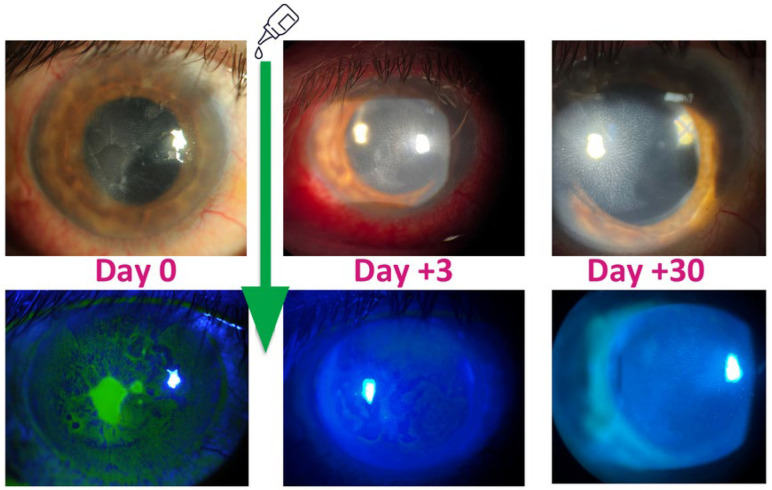
Images in the top row depict the anterior segment with neurotrophic ulcer healing in a patient treated with insulin eye drops after a posterior vitrectomy. Images in the bottom row display the anterior segment of the eye, stained with fluorescein and illuminated under cobalt blue light. Source: Authors’ own resources.

In other studies, one to two insulin drops administered 4–6 times daily were recommended [7,21,30]. This is probably due to the pharmacodynamics of rapid-acting insulins when they are administered subcutaneously. This route of administration results in a peak in action around 3 h post administration, with effects completely abating after 8 h [31]. In studies conducted by Bastion et al. and Fai et al. [24], patients treated for refractory ulcers due to neutrophic keratophy with Actrapid insulin (Novo Nordisk, Denmark) (a rapid-acting recombinant DNA form of human insulin) experienced faster recovery than those receiving regular insulin in studies by Wang et al. [21] and Diaz-Valle et al. [30]. For the vast majority of patients, the cornea undergoes full epithelialization within several days to a few weeks. Every observed patient in mentioned studies showed a reduction in the ulcerative surface area [19,21,22,23,24,25,32]. The effectiveness of topical insulin use in the course of neurotrophic keratopathy is shown in Figure 1.

It is worth noting that only 5% of the administered drop dosage affects the corneal surface [33]. A significantly larger portion of the drug drains through the tear duct system instead of being absorbed by ocular tissues such as the cornea or conjunctiva. However, studies indicate that systemic drug absorption is negligible. In rat studies, ocular insulin drops did not influence blood glucose levels, and similarly, even doses as high as 100 IU/mL did not affect glycemia in healthy subjects [20,27,34].

## 4. Solvent and Storage

Insulin is typically dissolved in saline, but polyethylene glycol 400 (PEG 400) is favored over saline as a solvent for insulin in eye drops for several reasons. Currently, lubricating drops containing polyethylene glycol 400 are available as ready-to-use. Such solutions tend to be stable, have reduced risk of proteolysis, and have minimal potential for antigenicity and impact on the patient’s immune system [35,36]. Additionally, the polyethylene glycol in available lubricating drops can accelerate epithelial defect healing [37]. Lubricating drops are usually packaged in 10 mL bottles, and this volume is adequate for use of the compound drops within a month [19,22,23]. For preparation of the solution, choosing drops free of preservatives is advised because they might hinder the re-epithelialization process. In one rat study, a mixture with moxifloxacin was used [27]. Interactions between insulin and fluoroquinolone antibiotics during topical application are unknown, but systemically administered fluoroquinolones can cause dysglycemia; therefore, caution is recommended when treating diabetic patients [38].

The product characteristics card for rapid-acting insulin Humulin R (Eli Lilly, Indianapolis, IN, USA) includes the following storage recommendations [31]. When diluted, Humulin R maintains its properties for 28 days if stored at 5 °C, but only 14 days if stored at 30 °C. Additionally, intravenous infusion bags with HUMULIN R remain stable for 48 h at 2–8 °C. Afterward, they can be stored at room temperature for another 48 h. From these data, it has been inferred that once the medication is prepared, patients can use the drops for 28 days if the solution is stored in a refrigerator. If kept at room temperature, the medication should be used within 2 weeks. Containers that ensure proper sterility, such as minims or bottles with built-in filters, are essential [31]. Wang et al. suggested that the prepared medication should be used within 1 month of preparation [21]. Based on the drug’s shelf life, the volume of the preparation should suffice for the treatment duration.

## 5. Prescription Proposal

Based on our review of recent publications, insulin should be used at a low concentration of 0.5–1 IU/mL and dissolved in drops containing polyethylene glycol 400, which are typically commercially available in ready-to-use packaging. Depending on availability, a formulation without preservatives should be chosen. The container for patients can be in minims or packaging with a dropper that incorporates a built-in sterilizing filter [19,22,25]. This is a proposed formulation and should be adjusted based on the availability of preparations and the patient’s clinical condition.

## 6. Alternative Therapeutic Options in Corneal Ulcers due to Neutrophic Keratopathy

Currently, there are no published studies directly comparing the efficacy of insulin with cenegermin (Oxervate, Dompé, Milan, Italy), which is a recombinant human nerve growth factor (rhNGF). The outcomes of cenegermin use have been shown to be effective, even upon extended observation [39,40]. However, treatment with compound drops of insulin undeniably offers a more favorable economic profile. An 8-week course of Oxervate is priced around USD 100,000, whereas the cost of insulin drops, including potential pharmacy preparation expenses, is approximately USD 60–80 per bottle (Polish pharmacy quote for compound drug), which is adequate for a month’s therapy [41].

Insulin can be employed in the treatment of recurrent epithelial defects associated with neurotrophic keratopathy and following chemical burns. It is typically considered as a second-line treatment after the lack of improvement with autologous serum eye drops [24,26]. For neurotrophic ulcers after vitreoretinal procedures in diabetic individuals, insulin may be considered as a first-line therapeutic option [26].

## 7. Mechanism of Action

Based on a review of the literature, it can be concluded that insulin significantly accelerates the regenerative process within the cornea. Explaining this phenomenon is a challenge, as it is possible that many processes and metabolic pathways overlap. Researchers have attempted to explain this phenomenon. There are reports in the literature that topical insulin has a positive effect on cutaneous defects in both diabetic and non-diabetic rats [42].

Neurotrophic factors within the cornea, including leukemia inhibitory factor (LIF), glial cell-derived neurotrophic factor (GDNF), brain-derived neurotrophic factor (BDNF), neurotrophins 3, 4, and 5 (NT-3/4/5), nerve growth factor (NGF), ciliary neurotrophic factor (CNTF), epidermal growth factor (EGF), hepatocyte growth factor (HGF), keratinocyte growth factor (KGF), and notably, insulin-like growth factor-1 (IGF-1) along with insulin-like growth factor binding protein-3 (IGFBP-3), are synthesized by epithelial cells and fibroblasts. These factors are pivotal in controlling cellular processes such as migration, proliferation, differentiation, and survival.

Insulin, in conjunction with peptide ligands insulin-like growth factor (IGF)-1 and IGF-2, orchestrates the functional mechanism of the IGF system. Insulin, structurally similar to IGF-1 and present within the tear film, also plays a role in cellular growth and corneal repair, activating the ERK and PI-3K pathways within corneal epithelial cells. These pathways, particularly PI-3K, facilitate the Akt signaling route, which is crucial for their anti-apoptotic properties. In conditions such as hypoxia and hyperglycemia, the expression of IGFBP-3 is amplified, a phenomenon also noted in the tears of individuals with diabetes, where it is associated with changes in the sub-basal nerve plexus and the basal layer of corneal epithelial cells. Stressful conditions lead to a decrease in IGFBP-3, while IGF-1 receptor expression is elevated, aiming to promote cell growth and survival. In the context of diabetes, the dysregulation characterized by heightened basal IGFBP-3 and diminished SIRT1 (Sirtuin 1) levels contributes to compromised corneal epithelial healing [9].

### 7.1. Receptors

The insulin-like growth factor family of ligands (IGF-I, IGF-II, and insulin), receptors, and IGF-binding proteins are crucial participants in both human physiology and the process of tissue repair. So far, six IGFBP proteins have been recognized. They are responsible for preventing IGF-1-induced IGF-1R activation by binding to IGF-1 to prolong its half-life in the circulation. The insulin-like growth factor family plays a pivotal role in maintaining corneal integrity, as well as cell proliferation, migration, and metabolic regulation.

Receptors for IGF type 1 (IGF-1R), IGF type 2 (IGF-2R), insulin receptor (INSR), and the hybrid of IGF-1R and INSR (Hybrid-R) are present in the cornea. These receptors have different structures. For example, IGF-1R and INSR are similar to each other. They are transmembrane glycoproteins consisting of two beta subunits, each incorporating an intracellular tyrosine kinase domain, and two alpha subunits each with an extracellular ligand-binding domain. IGF-2R, on the other hand, is a monomeric protein with 15 different extracellular domains.

These receptors also occur intracellularly, performing specific roles. IGF-1R, INSR, and their hybrid molecule influence mitochondrial stability and also play a role in the expression of nuclear genes. IGF-1 and IGF-2 are among the most important polypeptide growth factors that act in all cellular layers of the cornea [5].

**Figure 2:**
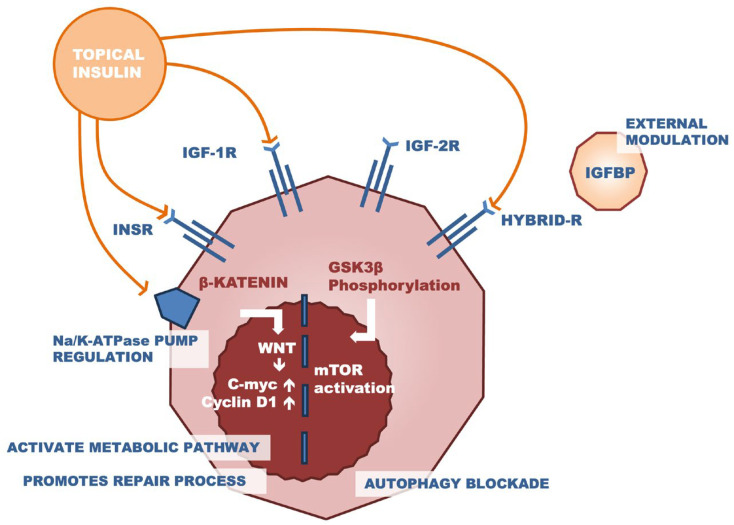
Illustration of the effect of insulin on the wound-healing process. The detailed mechanism of action is presented in the text.

Receptors for IGF type 1 (IGF-1R), IGF type 2 (IGF-2R), insulin receptor (INSR), and the hybrid of IGF-1R and INSR (Hybrid-R) are present in the cornea. These receptors play a pivotal role in maintaining corneal integrity, as well as cell proliferation, migration, and metabolic regulation. Glucose uptake occurs via an alternative route through the GLUT1 transporter. IGF-1R, INSR, and their hybrid molecule influence mitochondrial stability and also play a role in the expression of nuclear genes. External modulation is performed by insulin-like growth factor-binding proteins (IGFBP), which bind to IGF-1, extending its half-life and inhibiting the activation of the IGF-1R receptor (Figure 2) [5].

### 7.2. WNT/β-Catenin Pathway

Nakatsu et al. [6] conducted a study on the Wnt/β-catenin pathway in human corneal stem cells (LSCs). They isolated total RNA from the human corneal limbus and central cornea, followed by a quantitative real-time PCR reaction to identify transcripts specific to each region. Wnt protein expression was detected using an immunohistochemical method. The study revealed an increased expression of Wnt genes in the corneal limbus region compared to the central cornea. The activation of Wnt/β-catenin signaling was found to promote the proliferation of corneal stem cells. Confocal microscopy analysis showed the presence of β-catenin on the plasma membrane in all epithelial cells, with minimal detection in the cytoplasm and nucleus, and the least in corneal limbus epithelial cells in the basal and suprabasal layers. Various genes related to Wnt signaling were preferentially expressed in either the human corneal limbus or cornea. The researchers concluded that the Wnt signaling pathway is present in ocular surface epithelial cells, with several Wnt activators and inhibitors exclusively found in the corneal epithelial stem cell region. This discovery suggests a role for Wnt signaling in regulating corneal epithelial cell proliferation [6].

Yang et al. [43], utilizing cultured TKE2 cells and trigeminal ganglion neurons, investigated the interaction of insulin on the cornea in vitro and the corneal wound healing process in diabetic mice in vivo. Both insulin and Wnt signaling pathways play roles in cell proliferation, tissue regeneration, and homeostasis. The researchers proposed that topically applied insulin binds to the insulin receptor, activating insulin signaling pathways and influencing the accumulation of β-catenin in the cell cytoplasm. Subsequently, β-catenin is transported to the cell nucleus, where it activates the Wnt signaling pathway, increasing the transcription and translation of genes c-Myc, cyclin D1, and Tcf4, which affect the regenerative and reparative processes of the corneal epithelium and nerve cells in diabetic mice [5]. It is presumed that this process also occurs in the cornea, but this still requires confirmation.

### 7.3. PI3K/AKT Pathway

It has been established that insulin affects the activation of the phosphatidylinositol 3-kinase(PI3K)/Akt/mTOR signaling pathway. Insulin binds to its receptor—INSR or IGF-1R on the cell membrane, activating metabolic pathways [5].

Researchers have shown that insulin regulates Akt phosphorylation at ser473 in human corneal epithelial cells, leading to an increase in GSK3β phosphorylation and resulting in mTOR activation and autophagy blockade. Insulin also inhibits the processes of autophagy and mitochondrial destruction—mitophagy [5].

The PI3K/AKT pathway influences several metabolic processes, cell proliferation, and the cell cycle, as well as impacting telomerase activity. Apoptosis, inflammation, and the presence and concentration of reactive oxygen species (ROS) are also affected.

Animal studies have shown that the local application of insulin improves both corneal wound healing and increases corneal sensation. It is believed that this may occur through the action of insulin on its receptor and the IGF-1R receptor, influencing the activation of the PI3K pathway. Peterson and Chandler conducted a study focusing on this mechanism. They assessed the effect of corneal repair processes under various glucose concentrations, depending on the presence of insulin in vitro.

The researchers conducted studies on corneal cell cultures derived from human and canine tissues. They used an immortalized line of human telomerized corneal epithelial cells (hTCEpi), canine corneal epithelial cells (CEC), stromal fibroblasts, and stromal isolates from human and canine cadavers. The study showed that in the culture of human corneal epithelial cells and the absence of additional glucose, the regenerative process with insulin was significantly more effective (*p* = 0.0004) compared to the control sample without insulin. It was also demonstrated that high glucose concentrations negatively affected the repair process. In trials comparing wound healing involving human stromal fibroblasts, insulin treatment resulted in significantly better effects (*p* ≤ 0.001) compared to the control group. The decrease in PI3K expression significantly correlated with an increase in glucose levels in both: human CECs treated with and without insulin (*p* ≤ 0.0001).

The conducted studies confirmed that insulin did not cause negative changes and did not induce cytotoxicity. The researchers believe that insulin, upon binding to its receptor and the IGF-1 receptor, induces activation of the receptor tyrosine kinase (RTK), which, by causing phosphorylation, interacts with the insulin receptor substrate (IRS). These changes affect the PI3K pathway, translating into the promotion of processes that promote cell proliferation and migration through phosphorylated Akt (pAkt) and mTOR (p-mTOR). Further research is necessary to delve into this issue and explore interspecies differences [5,42].

### 7.4. Na/K-ATPase Pump and Insulin

The Na/K-ATPase pump is responsible for maintaining a balanced, normal level of corneal stroma hydration. Insulin also plays a role in regulating this process by enhancing the activity of Na/K-ATPase. The reduction in the number of endothelial cells can lead to dysfunction, interfering with the normal function of this pump. Insulin exhibits plei-otropic effects in repair processes and the maintenance of corneal homeostasis. Due to the complexity of the mechanisms involved, further studies are needed to clarify the met-abolic pathways it affects [44].

## 8. Conclusions

Topical administration of rapid-acting insulin eye drops is an effective and safe alternative for the treatment of difficult epithelialization disorders. The concentration and frequency of administration should be tailored to the severity of the local condition and adjusted based on the initial therapeutic response. Improvement is usually rapid. Although insulin has an important role to play in the treatment of corneal epitheliopathies, therapies (anti-glaucoma, antibiotic, anti-inflammatory) that do not adversely affect corneal re-epithelialization should not be overlooked. Further research is essential to elucidate the precise mechanism of action of topical insulin in healing ulcers and epithelial defects and to determine the optimal concentration of insulin in eye drops for the treatment of epithelial damage. A double-blind study comparing the efficacy of insulin and cenegermin would be valuable. This would require multi-center collaboration due to the rare occurrence of neurotrophic keratopathy.

## Figures and Tables

**Table 1:** Causes of neurotrophic keratopathy [8,9,10,11,12,13].

Causes of Neurotrophic Keratopathy
**(1) Genetic Causes:**
(a) Riley–Day syndrome (hereditary sensory neuropathy type III, familial dysautonomia)
(b) Möbius syndrome
(c) Familial corneal hypoesthesia (familial trigeminal anesthesia)
**(2) Systemic Diseases:**
(a) Diabetes mellitus
(b) Leprosy
(c) Vitamin A deficiency
(d) Amyloidosis
(e) Multiple sclerosis
**(3) Central Nervous System Diseases:**
(a) Intracerebral tumors
(b) Postoperative, particularly after brainstem surgery
(c) Aneurysm
(d) Cerebrovascular diseases of the brainstem
(e) Stroke (brainstem)
(f) Degenerative CNS diseases (Alzheimer’s disease, Parkinson’s disease)
(g) Acoustic neuroma
(h) Trigeminal neuralgia
(i) Other surgical injuries to the trigeminal nerve
**(4) Ocular Causes:**
(a) Corneal infections:
(i) Herpes infections (Herpes simplex and Herpes zoster)
(ii) Other infections, e.g., Acanthamoeba
(b) Chemical burns
(c) Side effects of topical medications and their conversants:
(i) Benzalkonium chloride (BAK), Timolol, Betaxolol, Sulfacetamid 30%, Trifluridine, Diclofenac
(ii) Abuse of local anesthetics
(d) Diseases comprising ocular surface disorders:
(i) Ocular surface Chronic severe blepharitis and/or rosacea
(ii) Entropion
(iii) Ocular injuries or chemical burns Graft-versus-host disease
(iv) Sjögren’s syndrome
(v) Ocular cicatricial pemphigoid
(vi) Trachoma
(e) Eye surgeries:
(i) Cataract surgery
i. Photorefractive keratectomy (PRK) and Laser in situ keratomileusis (LASIK)
ii. Penetrating keratoplasty and deep anterior lamellar keratoplasty (DALK)
iii. Collagen cross-linking for the treatment of keratoconus
iv. Following excessive laser coagulation of the retina or ciliary body as Pan-retinal photocoagulation or during vitrectomy
v. Multiple ocular surgeries
(f) Other ocular causes:
i. Contact lens use
ii. Orbital tumors
iii. Corneal dystrophies
iv. Radiotherapy sequelae

**Table 2:** Causes of neurotrophic keratopathy in order of highest frequency [8,9,10,11,12,13].

Causes of Neurotrophic Keratopathy (In Order of Highest Frequency)
(1) Iatrogenic, pars-plana vitrectomy
(2) Chronic ocular surface disease
(3) Ocular infections, especially herpetic
(4) Diabetes mellitus
(5) Central nervous system disease

## Data Availability

The data presented in this study are available in this article.
